# Collated data of mutation frequencies and associated genetic variants of bedaquiline, clofazimine and linezolid resistance in *Mycobacterium tuberculosis*

**DOI:** 10.1016/j.dib.2018.09.057

**Published:** 2018-09-24

**Authors:** N. Ismail, S.V. Omar, N.A. Ismail, R.P.H. Peters

**Affiliations:** a Department of Medical Microbiology, Faculty of Health Sciences, University of Pretoria, 0002 Prinshof, Gauteng, South Africa; b Centre for Tuberculosis, National Institute for Communicable Diseases, National Health Laboratory Service, Sandringham, Gauteng, South Africa; c Department of Medical Microbiology, School CAPHRI (Care and Public Health Research Institute), Maastricht University, the Netherlands

## Abstract

A comprehensive literature search was conducted to obtain previously published resistance associated mutations for bedaquiline, clofazimine and linezolid for *Mycobacterium tuberculosis*. Where possible, mutation frequencies for these three drugs were also identified. This catalog of previously published mutations could serve as a reference for comparing mutations associated with either in vitro or clinical resistant mutants. The usage of these data was seen in our study relating to approaches for resistance mutant creation (in vitro approaches for generation of *Mycobacterium tuberculosis* mutants resistant to bedaquiline, clofazimine or linezolid and identification of associated genetic variants (Ismail et al., 2018 in press). Previously published mutations for clofazimine were described in the *rv0678* and *rv1979c* genes, for bedaquiline in *atpE*, *rv0678* and *rv2535c* (*pepQ*) genes and for linezolid in the *rplC* and *rrl* genes.

**Specifications table**

**Table:** 

Subject area	Biology
More specific subject area	Microbiology
Type of data	Tables
How data was acquired	Literature search for mutation frequencies and genetic variants related to bedaquiline, clofazimine and linezolid resistance
Data format	Filtered
Experimental factors	Published articles regarding bedaquiline, clofazimine and linezolid resistant isolates and associated mutations
Experimental features	Previously published mutations in *rv0678*, *rv1979c*, *rv2535c*, *atpE*, *rplC* and *rrl* genes and mutation frequencies associated with either bedaquiline-, clofazimine- or linezolid-resistant *M. tuberculosis*
Data source location	South Africa
Data accessibility	Data is included in this article and accessible in related referenced articles
Related research article	Ismail, N., Omar, S.V., Ismail, N.A., Peters, R.P.H. (2018). in vitro approaches for generation of *Mycobacterium tuberculosis* mutants resistant to bedaquiline, clofazimine or linezolid and identification of associated genetic variants, JMM (In press)

**Value of the data**

• The data pertaining to genetic variants for bedaquiline, clofazimine and linezolid resistance are vital for understanding drug mechanisms of action.
• A catalog such as this may prevent data replication and serve as a comparator or reference for other studies related to resistance-associated mutation.
• The combined data of the resistance-associated variants could provide a starting point for the design of molecular susceptibility tests.

## 1 Data

The data in this article includes previously published mutations identified in both in vitro (Tables 1 and 3) as well as clinical isolates (Tables 2 and 4) associated with bedaquiline, clofazimine or linezolid resistance. Data were filtered according to the type of mutation identified and the gene in which the mutation occurred. Where available the in vitro approach used to generate the mutant, any information around the strain the mutants were derived from, the mutant MIC value as well as the article from which the data was derived from were included in the data. As bedaquiline- and clofazimine-resistant isolates tend to harbor *rv0678* mutations, data for these mutants were presented together. The final data table (Table 5) describes previously published mutation frequencies. A diversity of mutations were identified in the *rv0678* gene and were scattered along the gene. For the *atpE* gene, hotspots have been identified at positions 28 and 63. Mutations in the *rplC* and *rrl* target genes appear to be associated with either high or low-level linezolid resistance respectively. Four mutations in *rv2535c* were identified in in vivo bedaquiline and clofazimine resistant isolates. *Rv1979c* mutations were found in clinical pre-XDR and XDR isolates.

**Table 1:** Catalog of previously published mutations from bedaquiline- and clofazimine-resistant in vitro and in vivo isolates.

Mutation			Approach	Notes	MIC (µg/mL)		Refs.
*rv0678*	*atpE*	*rv2535c*			BDQ	CFZ	
–	G187C	–	Serial Passage	Fully susceptible strain	>8	–	[1]
T461C	A83G			Isoniazid-resistant strain	>8		
201_206del	A83C			Kanamycin-resistant strain	>8		
–	G183T			Pyrazinamide-resistant strain	>8		
	A83G						
A63T	A83G			Rifampicin-resistant strain	>8		
G74A	–			Fully susceptible strain	–	4	
T131C				Isoniazid-resistant strain		>4	
T407C				Kanamycin-resistant strain		4	
C204A				Pyrazinamide-resistant strain		>4	
T131C				Rifampicin-resistant strain		4	
–	A83T	–	Spontaneous	Fully susceptible strain	>8	–	
C403G				Pyrazinamide-resistant strain	4		
–	A83G			Pyrazinamide-resistant strain	8		
–	G187C			Pyrazinamide-resistant strain	>8		
193delG	–			Fully susceptible strain	–	2	
193delG				Fully susceptible strain		4	
A65T				Fully susceptible strain		4	
T407C				Pyrazinamide-resistant strain		1	
C214T				Pyrazinamide-resistant strain		2	
G137A				Pyrazinamide-resistant strain		4	
A97G							
–	–	CinArg271	in vivo	Mice treated with BDQ only	0.12	0.5-1	[2]
		+CinAla14		Mice treated with BDQ and CFZ	0.12		
		+CinAla14			0.12		
		L44P			0.12		
A413G	WT	–	in vitro mutants	Mutants derived from H37Rv	0.25	–	[3], [5]
G281A	WT				0.5		
A202G	WT			Mutants derived from MDR *M. tuberculosis* clinical strain	0.5		
Ins G 192-193	I66M				4		
IS6110 nt 272	WT				1		
Ins A 38–39	WT				1		
–	A95T	–	Mutagenesis	*M. smegmatis*	–	–	[4], [5]
–	C198G	–	Spontaneous	*M. tuberculosis*	–	–	[6]
	G187C				–	–	
–	G183T	–	Spontaneous	2 isolates from MDR strain	0.24–0.48	–	[7]
	G187C			3 isolates from MDR strain	0.9–3.84		
	A83T			1 isolate from MDR strain	0.48		
	C198G			1 isolate from MDR strain	0.48		
	A83G			1 isolate from WT strain	0.3		
	G183T			3 isolates from WT strain	0.48–0.96		
	G187C			4 isolates from WT strain	0.24–0.9		
–	G187C	–	Spontaneous	–	4–8x MIC	–	[8]
	A95T				4–8x MIC		
C189A	–	–	Spontaneous	–	0.5	1.25	[9]
C400T					0.5	1.25	
G193 deletion	–	–	Spontaneous	23 isolates	–	All ≤1	[10]
G193 insertion				21 isolates			
C466T				11 isolates			
C364 insertion				5 isolates			
A202G				5 isolates			
T2C				2 isolates			
G58T				1 isolate			
C107T				1 isolate			
G125A				1 isolate			
T29 insertion				1 isolate			
C98A				1 isolate			
T128G				1 isolate			
G137A				2 isolate			
A152G				1 isolate			
C158T				1 isolate			
C176T				1 isolate			
G188A				1 isolate			
G194A				2 isolates			
G197T				1 isolate			
C226T				1 isolate			
C251A				1 isolate			
G266T				1 isolate			
G269C				1 isolate			
A292 deletion				1 isolate			
G304A				1 isolate			
C305T				2 isolates			
T341C				1 isolate			
T365C				1 isolate			
CGCTGGGC371–378 deletion				1 isolate			
CG444–445 deletion				1 isolate			
G193 insertion				1 isolate			
–				3 isolates			
–		G265T		1 isolate			
–	A63P	–	–	Mutants from H37Rv reference strain	4	–	[11]
	D28G			Mutants from *M. tuberculosis* clinical isolates	0.5		
	E61D				0.5		
	L59V				0.25		
	I66M				1		

Mutations described in *rv0678*, *atpE* and *rv2535c* genes. A dash (–) is used to indicate where no data is available. WT-wild type, no variants detected. BDQ-Bedaquiline. CFZ-Clofazimine.

**Table 2:** Catalog of previously published mutations from bedaquiline- and clofazimine-resistant clinical isolates.

**Mutation**			**Notes**	**MIC (µg/ml)**		**Refs.**
***rv0678***	***atpE***	***rv1979c***		**BDQ**	**CFZ**	
T124C	–	–	Clinical strains from BDQ trial	0.25		[3]
A97C				0.5	–	
C107T				0.5		
Del C 212				0.5		
Ins IS6110 nt 272				0.5		
Ins C 141–142				0.25		
2T>C	WT	–	fMet1Ala-relapse isolate after	0.5	4	[12], [13], [14]
			BDQ compassionate use			
T437C	WT	WT	XDR	0.78	1.2	[15]
G5T	WT	WT	Pre-XDR	0.73	4	
C158T	WT	WT	Pre-XDR	0.39	2.09	
T350G	WT	WT	XDR	1.54	4.16	
WT	WT	A155C	Pre-XDR	0.08	1.2	
Del gg 18–19	–	–	MDR isolates	0.5	–	[16]
Ins G140				0.25		
M139T				0.25		
198–199 Ins G	–	–	Mix: WT + *rv0678* mutant	0.24;0.48;1	–	[17], [18]
274–275 Ins A				1		
C148T, A187G			intergenic mutation, *rv0678* mutant	0.48		
G334C, (-13) Ins IS6110				0.48		
C185T				0.48		
C155T				0.48		
C176T				0.48		
224–225 Ins A				0.24		
T(-44)C				0.24		
A263G			Mix: WT + *rv0678* mutant	0.12		
T116C				0.12		
T124C			Mix: WT + *rv0678* mutant (silent mutation)	0.12		
C45T				0.12		
G256A				0.12		
[Ins139g]	WT	–		0.12–0.25	–	[19]
L142R	WT		Baseline and post-treatment BDQ isolates from BDQ clinical trials	0.25		
L142R	A63V			0.25–1		
[Del198G] [Del212C] [G233C, G78A]	WT			0.12		
[G66W] [Del198G] [Ins263A] [Del435T]	WT			0.12		
[Del198G] [Ins466C]	WT			0.25		
[Del435T]	WT			0.25		
[E113K] [Del198G] [Del435T]	WT			0.25		
[Del435T]	WT			0.25		
G121E	WT			0.25		
[L40S] [Del291C] [Ins386C]	WT			0.25		
Del291C	WT			0.25		
[S53P] [Del198G] [Del336C]	WT			0.25		
M23L	WT			0.06		
M23L Ins142C	WT			0.12		
M23L [Ins142C] [Ins419G]	WT			0.12		
M23L Ins419g	WT			0.12		
[Del19G] [E49stop] [Del198G] [Ins468GA]	WT			0.12		
-[V85A] [R135W]	WT			0.12		
V85A	WT			0.12		
Ins44A	WT			0.06		
[Ins144C]	WT			0.12–0.25		
Ins421G	WT			0.12–0.25		
Del32G	WT			0.06; 0.25		
[Y26stop] [L122P]	WT			0.12		
L122P	WT			0.12		
[Del214C] [Del198G]	WT			0.06		
[F79S] [Ins137G]	WT			0.12		
[Del19G] [Del198G]	WT			0.12		
A98V	WT			0.12–0.25		
WT	D28N			0.12		
[Ins139G] + [Ins318CG]	WT			0.12		
[Del274–283] [Ins139TG]	WT			0.12		
[C46R] [Ins139TG] [L40S]	WT			0.12		
Ser53Pro	WT	–	2 XDR isolates	0.5	2–4	[20]
Ser53Leu			1 XDR isolate	0.25	2	
Tyr157Asp			1 XDR isolate	0.125	2	
WT			1 XDR isolate	0.5	2	
WT	WT	G1226A	3 XDR isolates: Culture negative at 6 months	0.25–1 (MGIT) 0.06–0.125 (BMD)	0.5 (MGIT)	[21]
136_137insG		G1226A	XDR: : Culture positive at 6 months	2 (MGIT) 0.25 (BMD)	0.5 (MGIT)	
138_139insG		G1226A	XDR: Culture positive at 6 months	2 (MGIT) 0.5 (BMD)	2 (MGIT)	
141_142insC		G1226A	2 XDR isolates: Culture positive at 6 months	4 (MGIT) 0.25–0.5 (BMD)	0.5–1 (MGIT)	
T200G		G1226A	XDR: Culture positive at 6 months	4 (MGIT) 0.5 (BMD)	2 (MGIT)	
345delG		G1226A	XDR: Culture positive at 6 months	4 (MGIT) 0.5 (BMD)	1 (MGIT)	
-11C>A	WT	–	Fully susceptible clinical isolate	0.016	–	[22]
D5G	WT		Fully susceptible clinical isolate	0.016		
M23V	WT		STR resistant clinical isolate	0.063		
D47fs	WT		XDR clinical isolate	0.5		
E55D	WT		Fully susceptible clinical isolate	0.063		
G87R	WT		Fully susceptible clinical isolate	0.063		
R96Q	WT		INH resistant	0.25		
L117R	WT		Fully susceptible clinical isolate	0.016		
WT	-53G>A		Fully susceptible clinical isolate	0.125		
WT	-72T>C		RIF and INH resistant clinical isolate	8		
WT	-138T>C		3 RIF and INH resistant clinical isolates	0.031		
WT	183G>A		Fully susceptible clinical isolate	0.063		
WT	I66V		Fully susceptible clinical isolate	0.125		

Mutations described in *rv0678*, *atpE* and *rv1979c* genes. A dash (–) is used to indicate where no data was available. WT-wild type, no variants detected. MGIT- MGIT960 platform used to determine MIC. BMD- Broth Micro Dilution method used to determine MIC. BDQ-Bedaquiline. CFZ-Clofazimine.

**Table 3:** Catalog of previously published mutations from linezolid-resistant in vitro mutants.

**Mutation**		**Approach**	**Note**	**MIC value (µg/ml)**	**Refs.**
***rrl***	***rplC***				
–	T460C	Serial passage	Isoniazid-resistant strain	>8	[1]
	T460C		Kanamycin-resistant strain	8	
	T460C		Pyrazinamide-resistant strain	>8	
	T460C		Rifampicin-resistant strain	8	
–	T460C	Spontaneous	Fully susceptible strain	>8	
G2270C	–		Pyrazinamide-resistant strain (13 mutants derived)	4	
A2810C	–			4	
–	T460C			8 to >8	
G2061T	–	Spontaneous	4 isolates	32	[23], [24]
G2576T	–		1 isolate	16	
none	–		5 isolates	4-8	
C2848A	–	Serial passage	17 of 32 had *rrl* mutations, remainder had *rplC*	–	[25]
A2810T	–				
G2270C	–				
G2270T	–				
G2746A	–				
–	T460C				
–	T460C	Spontaneous	3 in vitro mutants selected for sequencing	16	[26]
	T460C			32	
G2270T	–			8	
G2814T	–	Spontaneous	4 isolates	25–50	[27]
–	T460C		12 isolates	50	
G2299T	–		7 isolates	65–156	
A2689T	–		1 isolate	60	
G2814T	–		4 isolates	94	

Mutations described in *rrl* and *rplC* genes. A dash (–) is used to indicate where data was not available.

**Table 4:** Catalog of previously published mutations from linezolid-resistant clinical isolates.

**Mutation**		**Note**	**MIC value (µg/ml)**	**Refs.**
***rrl***	***rplC***			
WT	–	No mutations in *rplD*, *rplV*, *whiB7*, *rrl*, *erm-*37	8 (3 strains)	[23]
WT			4 (1 strain)	
–	T460C	2 resistant, 3 acquired resistance during treatment	4–16	[24]
G2447T	–	acquired resistance during treatment	16	
–	T460C	–	2	[28]
–	T460C		0.5	
–	T460C		4	
G2576T	–		4	
G2576T	–		4	
–	T460C	8 isolates	–	[19]
G2814T	–	1 isolate		
C1921T	–	1 isolate		
G2294A	–	1 isolate acquired resistance during treatment		
G2576T	–	1 MDR-TB isolate	4	[13]
A2572C	–			
–	T460C	2 XDR-TB isolates	4–16	[12]

Mutations described in *rrl* and *rplC* genes. A dash (–) is used to indicate where data was not available. WT- wild type, no variants detected.

**Table 5:** Mutation frequencies and selection concentrations for bedaquiline-, clofazimine- and linezolid-resistant spontaneous mutants from previously published studies.

**Drug**	**Conc (µg/ml)**	**Strain**	**Mutation frequency**	**Refs.**
Bedaquiline	0.12	Fully susceptible *M. tuberculosis*	5 × 10^–7^	[8]
	0.12	*M. smegmatis*	2 × 10^–8^	
	0.24	Fully susceptible *M. tuberculosis*	5 × 10^–8^	
	0.24	*M. smegmatis*	1 × 10^–8^	
	0.03-0.05	Fully susceptible *M. tuberculosis*	1 × 10^–8^	[11]
		Clinical *M. tuberculosis*	5 × 10^–8^	
		Clinical *M. tuberculosis*	1 × 10^–8^	
		Clinical *M. tuberculosis*	1 × 10^–8^	
	0.015-0.5	Clinical strain *M. fortuitum*	1.5 × 10^–8^	
	0.25-8	Clinical strain *M. abscessus*	1.5 × 10^–8^	
	1	Fully susceptible *M. tuberculosis*	6 × 10^–9^	[1]
		Pyrazinamide-resistant *M. tuberculosis*	4 × 10^−7^	
Clofazimine	0.25	Fully susceptible *M. tuberculosis*	5 × 10^−^^6^	[10]
	1	Fully susceptible *M. tuberculosis*	5 × 10^−^^5^	[1]
		Pyrazinamide-resistant *M. tuberculosis*	7 × 10^−^^7^	
Linezolid	–	Fully susceptible *M. tuberculosis*	2 × 10^−8^	[23]
	–	Fully susceptible *M. tuberculosis*	5 × 10^−9^	
	2	Fully susceptible *M. tuberculosis*	1 × 10^−^^8^	[1]
		Pyrazinamide-resistant *M. tuberculosis*	1 × 10^−^^7^	

A dash (–) is used to indicate where data was not available.

## 2 Experimental design, materials and methods

A catalog was compiled of mutations observed for in vitro, in vivo and clinical M. tuberculosis strains resistant to bedaquiline, clofazimine and linezolid as reported in studies that were identified through a comprehensive literature search. This was done by searching for combinations of each drug (or drug class) together with terms like “resistant”, “resistance”, “mutant”, “mutations” as well as “*Mycobacterium tuberculosis*”. Related and citing articles were also reviewed. The articles were then analyzed on the basis of the approach used and the mutations documented. Mutations were delineated as arising from either in vivo or clinical and in vitro.

## References

[1] Ismail N., Omar S.V., Ismail N.A., Peters R.P.H. (2018). In vitro approaches for generation of Mycobacterium tuberculosis mutants resistant to bedaquiline, clofazimine or linezolid and identification of associated genetic variants. J. Microbiol. Methods.

[2] Almeida D., Ioerger T., Tyagi S., Li S.Y., Mdluli K., Andries K., Grosset J., Sacchettini J., Nuermberger E. (2016). Mutations in pepQ confer low-level resistance to bedaquiline and clofazimine in Mycobacterium tuberculosis. Antimicrob. Agents Chemother..

[3] Andries K., Villellas C., Coeck N., Thys K., Gevers T., Vranckx L., Lounis N., de Jong B.C., Koul A. (2014). Acquired resistance of Mycobacterium tuberculosis to bedaquiline. PLoS One.

[4] Hards K., Robson J.R., Berney M., Shaw L., Bald D., Koul A., Andries K., Cook G.M. (2015). Bactericidal mode of action of bedaquiline. J. Antimicrob. Chemother..

[5] Koul A., Dendouga N., Vergauwen K., Molenberghs B., Vranckx L., Willebrords R., Ristic Z., Lill H., Dorange I., Guillemont J., Bald D., Andries K. (2007). Diarylquinolines target subunit c of mycobacterial ATP synthase. Nat. Chem. Biol..

[6] Petrella S., Cambau E., Chauffour A., Andries K., Jarlier V., Sougakoff W. (2006). Genetic basis for natural and acquired resistance to the diarylquinoline R207910 in mycobacteria. Antimicrob. Agents Chemother..

[7] Huitric E., Verhasselt P., Koul A., Andries K., Hoffner S., Andersson D.I. (2010). Rates and mechanisms of resistance development in Mycobacterium tuberculosis to a novel diarylquinoline ATP synthase inhibitor. Antimicrob. Agents Chemother..

[8] Andries K., Verhasselt P., Guillemont J., Gohlmann H.W., Neefs J.M., Winkler H., Gestel J. Van, Timmerman P., Zhu M., Lee E., Williams P., de Chaffoy D., Huitric E., Hoffner S., Cambau E., Truffot-Pernot C., Lounis N., Jarlier V. (2005). A diarylquinoline drug active on the ATP synthase of Mycobacterium tuberculosis. Science.

[9] Hartkoorn R.C., Uplekar S., Cole S.T. (2014). Cross-resistance between clofazimine and bedaquiline through upregulation of MmpL5 in Mycobacterium tuberculosis. Antimicrob. Agents Chemother..

[10] Zhang S., Chen J., Cui P., Shi W., Zhang W., Zhang Y. (2015). Identification of novel mutations associated with clofazimine resistance in Mycobacterium tuberculosis. J. Antimicrob. Chemother..

[11] Segala E., Sougakoff W., Nevejans-Chauffour A., Jarlier V., Petrella S. (2012). New mutations in the mycobacterial ATP synthase: new insights into the binding of the diarylquinoline TMC207 to the ATP synthase C-ring structure. Antimicrob. Agents Chemother..

[12] Somoskovi A., Bruderer V., Homke R., Bloemberg G.V., Bottger E.C. (2015). A mutation associated with clofazimine and bedaquiline cross-resistance in MDR-TB following bedaquiline treatment. Eur. Respir. J..

[13] Bloemberg G.V., Keller P.M., Stucki D., Trauner A., Borrell S., Latshang T., Coscolla M., Rothe T., Hömke R., Ritter C., Feldmann J., Schulthess B., Gagneux S., Böttger E.C. (2015). Acquired resistance to bedaquiline and delamanid in therapy for tuberculosis. New Engl. J. Med..

[14] Hoffmann H., Kohl T.A., Hofmann-Thiel S., Merker M., Beckert P., Jaton K., Nedialkova L., Sahalchyk E., Rothe T., Keller P.M., Niemann S. (2016). Delamanid and bedaquiline resistance in Mycobacterium tuberculosis ancestral Beijing genotype causing extensively drug-resistant tuberculosis in a Tibetan refugee. Am. J. Respir. Crit. Care Med..

[15] Xu J., Wang B., Hu M., Huo F., Guo S., Jing W., Nuermberger E., Lu Y. (2017). Primary clofazimine and bedaquiline resistance among isolates from patients with multidrug-resistant tuberculosis. Antimicrob. Agents Chemother..

[16] Veziris N., Bernard C., Guglielmetti L., Le Du D., Marigot-Outtandy D., Jaspard M., Caumes E., Lerat I., Rioux C., Yazdanpanah Y., Tiotiu A., Lemaitre N., Brossier F., Jarlier V., Robert J., Sougakoff W., Aubry A., MyRMA C.N.R., the Tuberculosis Consilium of the C.N.R.M., MyRMA, C.N.R, Tuberculosis Consilium of the C.N.R.M. (2017). Rapid emergence of Mycobacterium tuberculosis bedaquiline resistance: lessons to avoid repeating past errors. Eur. Respir. J..

[17] Villellas C., Coeck N., Meehan C.J., Lounis N., de Jong B., Rigouts L., Andries K. (2017). Unexpected high prevalence of resistance-associated Rv0678 variants in MDR-TB patients without documented prior use of clofazimine or bedaquiline. J Antimicrob. Chemother..

[18] Pym A.S., Diacon A.H., Tang S.J., Conradie F., Danilovits M., Chuchottaworn C., Vasilyeva I., Andries K., Bakare N., De Marez T., Haxaire-Theeuwes M., Lounis N., Meyvisch P., Van Baelen B., van Heeswijk R.P., Dannemann B. (2016). Bedaquiline in the treatment of multidrug- and extensively drug-resistant tuberculosis. Eur. Respir. J..

[19] Zimenkov D.V., Nosova E.Y., Kulagina E.V., Antonova O.V., Arslanbaeva L.R., Isakova A.I., Krylova L.Y., Peretokina I.V., Makarova M.V., Safonova S.G., Borisov S.E., Gryadunov D.A. (2017). Examination of bedaquiline- and linezolid-resistant Mycobacterium tuberculosis isolates from the Moscow region. J. Antimicrob. Chemother..

[20] Pang Y., Zong Z., Huo F., Jing W., Ma Y., Dong L., Li Y., Zhao L., Fu Y., Huang H. (2017). in vitro drug susceptibility of bedaquiline, delamanid, linezolid, clofazimine, moxifloxacin, and gatifloxacin against extensively drug-resistant tuberculosis in Beijing, China. Antimicrob. Agents Chemother..

[21] Ismail N.A., Omar S.V., Joseph L., Govender N., Blows L., Ismail F., Koornhof H., Dreyer A.W., Kaniga K., Ndjeka N. (2018). Defining bedaquiline susceptibility, resistance, cross-resistance and associated genetic determinants: a retrospective Cohort study. EBioMedicine.

[22] Martinez E., Hennessy D., Jelfs P., Crighton T., Chen S.C.A., Sintchenko V. (2018). Mutations associated with in vitro resistance to bedaquiline in Mycobacterium tuberculosis isolates in Australia. Tuberculosis.

[23] Hillemann D., Rusch-Gerdes S., Richter E. (2008). In vitro-selected linezolid-resistant Mycobacterium tuberculosis mutants. Antimicrob. Agents Chemother..

[24] Beckert P., Hillemann D., Kohl T.A., Kalinowski J., Richter E., Niemann S., Feuerriegel S. (2012). rplC T460C identified as a dominant mutation in linezolid-resistant Mycobacterium tuberculosis strains. Antimicrob. Agents Chemother..

[25] Zhang S., Chen J., Cui P., Shi W., Shi X., Niu H., Chan D., Yew W.W., Zhang W., Zhang Y. (2016). Mycobacterium tuberculosis mutations associated with reduced susceptibility to linezolid. Antimicrob. Agents Chemother..

[26] Balasubramanian V., Solapure S., Iyer H., Ghosh A., Sharma S., Kaur P., Deepthi R., Subbulakshmi V., Ramya V., Ramachandran V., Balganesh M., Wright L., Melnick D., Butler S.L., Sambandamurthy V.K. (2014). Bactericidal activity and mechanism of action of AZD5847, a novel oxazolidinone for treatment of tuberculosis. Antimicrob. Agents Chemother..

[27] McNeil M.B., Dennison D.D., Shelton C.D., Parish T. (2017). in vitro Isolation and characterization of oxazolidinone-resistant Mycobacterium tuberculosis. Antimicrob. Agents Chemother..

[28] Lee M., Lee J., Carroll M.W., Choi H., Min S., Song T., Via L.E., Goldfeder L.C., Kang E., Jin B., Park H., Kwak H., Kim H., Jeon H.S., Jeong I., Joh J.S., Chen R.Y., Olivier K.N., Shaw P.A., Follmann D., Song S.D., Lee J.K., Lee D., Kim C.T., Dartois V., Park S.K., Cho S.N., Barry C.E. (2012). Linezolid for treatment of chronic extensively drug-resistant tuberculosis. New Engl. J. Med..

